# Alcohol Use and Gambling Associated with Impulsivity among a Swedish University Sample

**DOI:** 10.3390/ijerph19042436

**Published:** 2022-02-20

**Authors:** David Forsström, Alexander Rozental, Kristina Sundqvist

**Affiliations:** 1 Department of Psychology, Stockholm University, Albanovägen 12, 114 19 Stockholm, Sweden; kristina.sundqvist@su.se; 2 Centre for Psychiatry Research, Department of Clinical Neuroscience, Karolinska Institutet & Stockholm Health Care Services, Region Stockholm Norra Stationsgatan 69, 113 64 Stockholm, Sweden; alexander.rozental@psyk.uu.se; 3 Department of Psychology, Uppsala University, von Kramers Allé 1A, 751 42 Uppsala, Sweden; 4 Great Ormond Street Hospital Institute of Child Health, University College London, 30 Guilford Street, London WC1N 1EH, UK

**Keywords:** alcohol use, gambling, risk, impulsivity, Swedish university sample

## Abstract

Excessive alcohol use and gambling can have negative consequences. Across countries, the risk of excessive alcohol use is more common in university populations than in the general population. However, few studies have investigated the prevalence of both alcohol use and gambling in this group. This study explores these behaviours in a Swedish university setting. In addition, this study investigates how impulsivity affects alcohol use and gambling. In total, 794 Swedish students answered an online survey. Data were analysed using descriptive statistics to determine prevalence, and multinomial logistic regression was used to determine the contribution of impulsivity, age, and sex to alcohol use and gambling. Compared to the Swedish national prevalence, the prevalence was higher for excessive alcohol use, but the prevalence of gambling was at the same level or lower. High levels of impulsivity and male sex increased the risk of excessive alcohol use, while older age lowered the risk of excessive alcohol use and gambling. The results indicate that primarily young men could benefit from primary prevention in a university setting. Also, screening for impulsivity in men might be one way to identify risk groups in a university population.

## 1. Introduction

Problematic alcohol use is one of the most important factors contributing to global health burden and societal costs [1]. In addition, problem gambling is increasingly recognised as a public health issue, and harms from gambling affect health and, even at low risk levels, contributes to a loss of quality of life similar to the long-term consequences of, for example, moderate alcohol use disorder [2,3]. In Sweden, 16% of adults (18–64 years) are classified as hazardous alcohol consumers, which is defined as consuming 14 glasses of alcohol for males and 9 glasses for females per week [4]. As for problem gambling, 1.3% of the population aged 16–87 years experience gambling problems, and an additional 2.9% experience less serious sub-clinical problems (problem gambling was defined as a score over 8 (high-risk gambling), at-risk gambling as a score of 3 to 7 (medium risk), and sub-clinical gambling as a score of 1 to 2 of the Problem Gambling Severity Index, according to the Swedish Longitudinal Gambling Study) [5]. Both hazardous alcohol consumption and problem gambling are more common among younger people than among older people and more common among men than among women.

Alcohol use and other behaviours have been linked to impulsivity [6,7]. However, of all the personality traits associated with problematic alcohol use and gambling, traits related to impulsivity appear to show the most robust relations to both alcohol- and gambling-related problems [8]. Impulsivity has been defined as ‘a predisposition toward rapid, unplanned reactions to internal or external stimuli without regard to the negative consequences of these reactions to the impulsive individual or others’ [9] on p. 1784.

However, the relationship between drinking or gambling and impulsivity may be influenced by, as well as influence, several other factors. For example, a longitudinal study following a sample of 7th grade students found impulsivity to be associated with the age of gambling onset only for individuals with low socio-economic status [10]. Motives for gambling have also been found to mediate the relationship between impulsivity and problem gambling [11]. Another study found that the higher the self-reported impulsivity, the more the self-reported adverse consequences from gambling [12]. According to a review, impulsivity was also linked to a craving for alcohol [13]. Furthermore, impulsivity has been found to mediate the association between early life stressors (i.e., childhood trauma or abuse) and severity of alcohol dependence [14].

Being male is commonly associated with both high impulsivity and problematic alcohol use and gambling and has been found to both moderate and mediate the relationship between impulsivity and alcohol problems [15]. Benjet et al. [16] found that being male was one of the most significant risk factors when it comes to students’ hazardous drinking. In addition, men who take bigger risks, but not women, have been found to score higher on impulsivity than men in the general population, a characteristic that obviously makes men more susceptible to unhealthy gambling behaviours [12].

Impulsivity, however, is a complex construct that includes lack of perseverance (the tendency to not finish tasks), lack of planning (involving acting without thinking), sensation seeking (encompassing behaviour tendencies of trying new and exciting activities or sensations), and negative/positive urgency (representing the tendency to act rashly in response to strong negative/positive emotions) [17,18,19,20].Consequently, according to a meta-analysis of studies examining impulsivity as a risk factor for alcohol use, results vary considerably [21]. In addition, studies on gambling have generated diverse results. For example, one study found high impulsivity to be the only personality characteristic associated with all five addictive behaviours studied [22], but another study found that impulsivity was not a significant predictor of problem gambling when included in a regression with other measures of personality traits associated with risk [23]. However, a recent review and meta-analysis found a link between neuroticism (of which impulsivity is a component) and gambling [24]. A similar result between impulsivity and gambling was found [12].

Several studies have investigated university students’ alcohol use and gambling behaviours. One review found a high prevalence of alcohol use and problematic alcohol use among European students [25]. A review focused on Great Britain and Ireland reported high prevalence of alcohol use and problematic alcohol use among students [26]. Among Hong Kong students, the lifetime prevalence for problem was 14.7% [27]. Another study found a high prevalence of problem gambling in a student population [28]. On an overall level, one review found that the prevalence of pathological gambling among university students was 6.13% and that being male was associated with an increase in prevalence, whereas age was not [29]. Similar results are present in Swedish research for alcohol use and gambling. One study found that 87% of a sample consisting of Swedish university students engaged in risky alcohol use [30]. Similar results were found by a study by Andersson et al. [31]. Problem gambling was associated with higher levels of alcohol use, and an increase in alcohol use led to an increase in the risk for problem gambling for individuals between 16 and 24 years of age [32].

In sum, both impulsivity and problematic alcohol use and gambling are complex constructs with multi-facet interactions that need to be explored further, especially in university students samples. Specifically, research needs to fill the knowledge gap regarding these risky behaviours among Swedish university students. This study addresses these issues. Furthermore, there are currently no studies that address the risk level when it comes to both alcohol and gambling among Swedish university students. Our study attempts to fill that gap by addressing these two risk behaviours at the same time.

### Aim of the Study

This study explores the level of alcohol use and gambling problems in a sample of Swedish university students. This approach includes two sub-goals: to describe the prevalence of different levels of drinking and/or gambling associated with risk and harm and to compare impulsivity and sex differences between students who have no self-identified drinking and gambling problems and students that have self-identified alcohol and/or gambling problems associated with risk. The hypothesis is that students with problems related to drinking and/or gambling will experience a higher degree of self-rated impulsivity compared to students with no problems related to drinking and/or gambling.

Additionally, another aim is to test whether age acts as a mediator for impulsivity in relation to negative consequences for alcohol use and gambling. This includes if sex works as a moderator when it comes to the association between impulsivity and negative consequences from alcohol use and gambling.

## 2. Materials and Methods

### 2.1. Procedure

In October 2020, potential participants were recruited via advertisements from the Karolinska Institute’s (KI) communications office. The only inclusion criterion was that respondents had to be enrolled at a Swedish university. No exclusion criteria were used. Given that KI is mainly a medical university, other students were also recruited by forwarding information about the study to two more universities in Sweden as well as through posts on student forums on various social media platforms (e.g., Instagram, Facebook, Accindi, and LinkedIn), such as the official profiles for Swedish universities. Those interested in participating followed a link redirecting them to a website containing details about the purpose of the study, the procedure surrounding the survey, ethics, and the principal investigators. They also learned that the second author would provide them a recorded lecture on procrastination once the survey was completed as a reward for participating. Once providing informed consent using a checkbox, the students were forwarded to the survey, which was managed in LimeSurvey. All information and the survey itself were available in English and Swedish. On average (SD = 16), the survey took 21 min to complete, and the questions/measures always followed the same order (i.e., no randomisation of the self-report measures). All items needed to be completed to go on to the next page of the survey, with one self-report measure being presented per page. A progress bar on top of the screen (0–100%) provided a visual of how much of the survey the student had completed.

### 2.2. Measures

#### 2.2.1. Alcohol Use Disorders Identification Test

The Alcohol Use Disorders Identification Test (AUDIT) was originally developed by [33] as a short screening tool for assessing alcohol use. The self-report measure has 10 items—e.g., ‘How many standard drinks containing alcohol do you have on a typical day when drinking?’, ‘How often do you have a drink containing alcohol?’—and is rated on a scale of 0–4, with higher numbers indicating more problematic alcohol use. The last two items, however, use a scale that scores as 0, 2, and 4. The total score of the AUDIT ranges from 0 to 40. The Cronbach’s α (i.e., internal consistency) was 0.82 for a Swedish version of the test. For the sample used in this study, the Cronbach’s α was 0.82.

#### 2.2.2. Problem Gambling Severity Index

The Problem Gambling Severity Index (PGSI) was derived from the Canadian Problem Gambling Inventory, which measures the signs and consequences of problem gambling. The self-report has nine items—e.g., ‘Have you felt guilty about the way you gamble or what happens when you gamble?’ and ‘Have you needed to gamble with larger amounts of money to get the same feeling of excitement?’—and is scored on a 0–3 rating scale. The total score of the PGSI ranges from 0 to 27 (Ferris & Wynne, 2001): a score of 8 and over indicates a high risk and problem gambling; a score pf 3–7 indicates being at risk for problem gambling and can be seen as medium risk; a score of 1–2 indicates a low risk [5,34]. The PGSI has a test–retest reliability of 0.78 and Cronbach’s α of 0.84. For the sample used in this study, the Cronbach’s α was 0.87.

#### 2.2.3. Susceptibility to Temptation Scale

The Susceptibility to Temptation Scale (STS) is a measure of sensitivity to delay and impulsivity, originally developed by [35] in relation to research on procrastination. The self-report measure has 11 items—e.g., ‘It takes a lot for me to delay gratification’—and is rated on a scale of 1–5, with higher numbers reflecting greater impulsivity. The total score of the STS is 11–55. The self-report measures, however, do not have established cut-offs, but in a sample of individuals seeking treatment for procrastination, their average score was 42.02 (SD = 7.07) [36]. The STS has a one-factor solution referred to as impulsivity and has an internal consistency of 0.89 [35]. A Swedish psychometric evaluation was also carried out [36], obtaining an internal consistency of 0.87 and a similar factorial structure. The STS is positively correlated to procrastination (*r*s = 0.39–0.53), anxiety (*r* = 0.30), depression (*r* = 0.20) and negatively correlated to quality of life (*r* = −0.21) [36]. For the sample used in this study, the Cronbach’s α was 0.94.

### 2.3. Study Sample Characteristics

The total sample consisted of 794 respondents: 532 females (67%), 258 males (32.5%), and 4 individuals (0.5%) who categorised themselves as ‘other’. When it comes to age distribution, the mean age was 28.9 years (SD = 8.2), and the median age was 27 years. For women, the mean age was 29.4 years (SD = 8.8), and the median age was 27 years. For males the mean age was 27.8 years (SD = 6.7), and the median age was 26 years. There was a significant difference in age between females and males—*t*(788) = 2.67, *p* = 0.008. However, this difference was small and not considered to have any influence on the results of the study. For the individuals who identified their sex as ‘other’ (i.e., people who did not want to define or disclose their sex), the mean age was 27.8 years (SD = 8.46), and the median age was 28.5 years.

The mean number of university credits achieved was 194.0 (SD = 136.4)—for females 194.0 (SD = 136.4), and for males 189.5 (SD = 143.8)—with no significant difference between men and women, *t*(788) = 0.43, *p* = 0.67. For the category ‘other’, the mean number of credits was 206 (SD = 235.6). One full-time semester equals 30 credits (i.e., European Credit Transfer System).

### 2.4. Data Analysis

Descriptive statistics was used to describe the prevalence levels in the sample. Chi^2^ analysis was also carried out to examine the distribution of frequencies for men and women. Multinomial regression was performed using risk categories for both AUDIT and PGSI. Multinomial regression has been used in previous studies regarding alcohol and risk [37,38]. The following cut-offs regarding the risk for classifying PGSI scores were used: zero points equalled no risk; one to two points equalled low risk; three to seven points indicated medium risk; eight and above corresponded to high risk [5,34]. In this study, however, the two highest categories were collapsed into one category. This was done because there were few high-risk gamblers. A similar procedure was used for AUDIT: 0 indicated no risk, 1 to 7 indicated low risk, 8 to 14 indicated medium risk, above 15 indicated high risk and alcohol dependence. The medium and high-risk categories were collapsed into one category. After that, the highest risk level for AUDIT or PGSI was used as the dependent variable in regression analysis, e.g., having a medium/high risk on both instruments resulted in a medium/high risk in the analysis. However, having a low risk on PGSI and a medium/high risk on AUDIT resulted in a medium/high risk in the analysis and vice versa if a respondent had a higher risk on the PGSI and a lower risk on AUDIT.

A mediator analysis was carried out using the total score on the impulsivity measure and the total score on AUDIT, using age as a mediator. Furthermore, a moderator analysis with the same premise as for the mediator analysis was carried out but using sex as a moderator. An analysis of PGSI was not included due to the few cases endorsing negative consequences from gambling. All these analyses used the MEDMOD package in Jamovi.

Statistical analyses were performed using SPSS V.28, and multinomial regression and mediator analysis were performed using Jamovi 1.6.23.

## 3. Results

### 3.1. Levels of Alcohol Use and Gambling in the Sample (n = 794)

The prevalence of alcohol use associated with a medium risk was 17.8% and that associated with a high risk was 4.0%. For women, medium risk use was found for 15.4% of the sample, and high risk for 2.6%. For men, these numbers were slightly higher: 22.5% of men had medium risk, and 7% had a high risk. The prevalence of gambling problems (i.e., individuals who were at medium risk and at high risk) was 1.1%. Medium-risk gamblers constituted 0.5% and high-risk gamblers (most likely, individuals with gambling problems) accounted for 0.6%. For women, those numbers were 0.2% for medium-risk gamblers and 0.6% for individuals who were at high risk for gambling problems. For men, these numbers were 1.2% for individuals at medium risk and 0.8% for individuals at high risk for gambling problems. See Table 1 for an overview.

The Chi^2^ test showed a significant difference for different risk levels of AUDIT between men and women, *X*^2^(3, N = 790) = 17.26, *p* < 0.01. There was also a significant difference for the risk levels for PGSI. However, one assumption (the count in the cells were lower than five in 60% of the cases) was violated, thus a likelihood ratio was used instead of Chi^2^. The result was 16.59, *p* < 0.01.

### 3.2. Results of the Multinomial Regression

Table 2 presents the results of the multinomial regression. Being male and experiencing higher levels of impulsivity were associated with having more problems (medium and high risk) in terms of alcohol use and gambling (even though the number of medium- and high-risk gamblers was low). Being older was also associated with a lower risk level. None of the variables were significant for low-risk alcohol use, and None of the variables were significant for low-risk alcohol use and gambling associated with low risk in comparison with no risk of alcohol use and gambling (reference category).

### 3.3. Results from the Mediator and Moderator Analysis

A mediator analysis was performed to investigate the effects of impulsivity and age on the risk behaviours. Age showed a partial mediation below 20% for the total score on AUDIT (16.20%), indicating that age did not mediate impulsivity. Sex was not a moderator based on the nonsignificant value of *p* = 0.78 (see Table 3).

## 4. Discussion

This study evaluated the prevalence of alcohol use and gambling associated with risk in a Swedish university population and investigated how sex, age, and impulsivity influence the risk level of alcohol and gambling by means of multinomial regression.

The results showed that the prevalence of alcohol problems was higher in the participants than in the Swedish population in general [4]. This is line with previous studies [25]. Overall, the levels of risk related to gambling was lower than the national average [5]. More specifically, for women, the level of gambling associated with high risk was in line with the national prevalence level of 0.6%. However, the level of medium risk related to gambling was lower than the national average for both men and women. For men, the results regarding high-risk gambling are comparable with the national Swedish average. The findings from our study contradict previous results [29]. One plausible explanation for this might the fact that the majority of the participants were women who, in general, tend to gamble less.

The results from multinomial regression are in line with our hypothesis, indicating that being male and having higher levels of impulsivity is associated with higher levels of risk for alcohol use and gambling. The results from the mediator and moderator analysis indicated that age was only a partial mediator and that sex was not a moderator in relation to the consequences of alcohol use. A possible explanation for this might be that the participants were older and that a link between impulsivity and age is primarily observed in younger individuals. What may have been measured was impulsivity as an actual stable trait in the sample due to the older age of the respondents (the mean age in the sample was almost 29 years). University studies might not be accessible and/or interesting for individuals with a high level of trait impulsivity. The opposite might be true for the current sample, thus resulting in skewed results when it comes to impulsivity. In addition, the model showed that older age decreases the risk for alcohol use and gambling.

### 4.1. Practical Implications

Given the results when it comes to the prevalence of alcohol use and gambling associated with medium and high risk, one possible implication from a preventive standpoint would be to administer a general screening tool focused on the risk for alcohol use and gambling to newly admitted students or as a regular check-up of their health and wellbeing once every semester. Several of the Swedish universities included in the study have health care units that provide psychiatric services. Screening for alcohol and gambling could be part of their provision of healthcare services. Students who score in the clinical range would get feedback on their results and information on where to seek further assistance, such as at a student healthcare centre. Likewise, students who are experiencing risk and would like to start up with low-intensity support could be advised to use an internet-based intervention, which is already provided by Swedish universities [39,40] One way could be to use a similar set up as that currently being evaluated in Swedish workplace settings [41].

Furthermore, because younger age, being male, and impulsivity are related to having more problems with alcohol use and, to some extent, gambling, targeted screening and psychoeducational programs might be an alternative route for preventing further difficulties. This might be particularly relevant for educational settings that tend to attract students with these demographics. Targeted primary prevention aimed towards identified risk segments could be another way to help this group. Information campaigns regarding alcohol and gambling could be one way of decreasing risk in this group.

As a preventive effort based on sex, the level of problems, and impulsivity, tailored interventions could be offered to alleviate symptoms early on for students experiencing these problems. This should be done in order to minimise the negative consequences of these behaviours. Students experiencing behaviours associated with risk (i.e., alcohol use) have a lower academic achievement when it comes to university studies [42,43,44,45]. Preventive efforts that target this group are also recommended [42]. Several reviews have found that interventions aimed towards college students are successful in reducing alcohol use [46,47]. In addition, providing a preventive effort might be one way of altering potentially harmful trajectories that span a lifetime.

However, one important aspect when it comes to prevalence rates in the results is that vulnerable individuals of both sexes do engage in behaviours associated with risk. Although being male stands out as a risk factor, women may still need to be targeted by preventive efforts.

### 4.2. Limitations

This study has several limitations that need to be addressed when reviewing the results. First, there was no control of whether the respondents attended a Swedish university. However, all the information about the survey was distributed using accounts managed by universities. All the advertisements and information targeted university students and also conveyed the information that a respondent had to be enrolled at a Swedish university in order to be eligible to participate. In addition, data collection was made during the second semester of 2020 (i.e., during the second wave of the COVID-19 pandemic). Similar to other countries, Sweden imposed several recommendations to manage the spread of the virus, which meant no on-campus education. It is unclear whether this influenced the students’ responses to the self-report measures included in this study. Research indicates that stress levels increased and mental health was impaired among students in the United Kingdom following the COVID-19 pandemic [48]. However, Sweden did not enforce restrictions such as shutdown or total isolation at home, suggesting that students in this study may have been less affected. One study found that the academic achievement improved during the pandemic, which could indicate a decrease in alcohol use since the study also found that high use of alcohol was associated with lower academic performance [49] and another found that alcohol use increased [50]. Based on these two studies, it is hard to ascertain how COVID-19 might have influenced the results. This conclusion is supported by a review that found both an increase and a decrease regarding alcohol consumption in different groups [51]. Participants were recruited by advertisements and posts on social media platforms, which may attract a certain type of student. This may therefore impact the generalizability of the findings, in regard to those experiencing more severe problems with alcohol and gambling or who may be less representative of students in Sweden in general, in terms of their demographics.

A review of drinking in relation to the COVID-19 pandemic found that drinking increased in many countries [52]. Since the data collection was carried during the pandemic, the increase in drinking behaviour might have affected the results.

Another limitation of the study is that drug use was not investigated. Having scores from the Drug Use Disorders Identification Test [53] could have provided more insight into the levels of risk for the sample.

A self-report bias could also have influenced the results, since participation was voluntary. How this might have influenced the students’ responses is difficult to ascertain, which makes it difficult to generalise the results to other populations. In addition, one study found that it was hard to determine the level of intoxication for both the individual who drinks and an observer [54]. The size of the sample is another limitation. More answers might have increased the variance. In combination with the self-report bias, this is a drawback of the study. However, since the sample consisted of approximately 800 individuals, some inferences are still possible to make. Perceived loss and actual loss in gambling indicate that individuals that gamble cannot assess their level of gambling involvement [55]. This could be reflected in the answers given in PGSI, since individuals can have a hard time determining how much they gamble and what types of negative consequences they can endure. This also holds true for alcohol use. This can in turn have resulted in distorted levels of gambling and alcohol use.

### 4.3. Future Research

Future research should focus on considering all types of risk behaviours including drugs and add other behavioural addictions (e.g., gaming and pornography consumption). Furthermore, interventions targeting different ways to ameliorate the rate of impulsivity present in university populations should also be carried out and thoroughly researched.

More information about alcohol, gambling, and drug use in university populations is needed, and surveys targeting this population should be carried out. Longitudinal studies with large samples including several universities are also needed.

## 5. Conclusions

Being male, being younger, and having higher rates of impulsivity seem to be linked to risky alcohol behaviours. Primary preventions targeting groups with these characteristics could be implemented to lower the prevalence. Interventions, especially internet-based and/or group-based, could help students change behaviours that can lead to alcohol problems and have a negative effect on their studies and life situation in the short and long term.

## Figures and Tables

**Table 1:** Prevalence of different risk levels for alcohol use and gambling.

Level or Risk	Measure							
	AUDIT Total n (%)	AUDIT Women n (%)	AUDIT Men n (%)	AUDIT Other n (%)	PGSI Total n (%)	PGSI Women n (%)	PGSI Men n (%)	PGSI Other n (%)
No risk	177 (22.3%)	118 (22.2%)	58 (22.5%)	1 (25%)	751 (95.1%)	517 (97.2%)	234 (90.7%)	4 (100%)
Low risk	444 (55.9%)	318 (59.8%)	124 (48.1%)	2 (50%)	30 (3.8%)	11 (2.1%)	19 (7.4%)	-
Medium risk	141 (17.8%)	82 (15.4%)	58 (22.5%)	1 (25%)	4 (0.5%)	1 (0.2%)	3 (1.2%)	-
High risk	32 (4.0%)	14 (2.6%)	18 (7%)	-	5 (0.6%)	3 (0.6%)	2 (0.8%)	-
**Total**	794 (100%)	532 (100%)	258 (100%)	4 (100%)	794 (100%)	532 (100%)	258 (100%)	4 (100%)

**Table 2:** Results of the Multinomial Regression.

							95% CI	
Risk Level	Predictor	Estimate	SE	Z	*p*	OR	UL	LL
Low Risk (No Risk ^1^)	Intercept	0.29557	0.49047	0.603	0.547	1.344	0.514	3.514
	Age	0.01266	0.01118	1.133	0.257	1.013	0.991	1.035
	Gender	−0.08115	0.19914	−0.408	0.684	0.922	0.624	1.362
	STS Total ^2^	0.00995	0.00876	1.136	0.256	1.010	0.993	1.027
Medium/High Risk (No Risk)	Intercept	−0.78516	0.65667	−1.196	0.232	0.456	0.126	1.652
	Age	−0.03996	0.01617	−2.471	0.013	0.961	0.931	0.992
	Gender	0.53096	0.22991	2.309	0.021	1.701	1.084	2.669
	STS Total	0.04929	0.01121	4.399	<0.001	1.051	1.028	1.074

^1^ Reference category; ^2^ STS = Susceptibility to Temptation Scale.

**Table 3:** Moderation Estimates.

			95% Confidence Interval			
	Estimate	SE	Lower	Upper	Z	*p*
STStot	0.07461	0.0146	0.0460	0.1033	5.102	<0.001
Sex	1.20081	0.3278	0.5583	1.8433	3.663	<0.001
STStot × Sex	−0.00909	0.0318	−0.0714	0.0533	−0.286	0.775

## Data Availability

Data can be retrieved by contacting the first author.
